# Metabolome and Transcriptome Analysis Revealed the Pivotal Role of Exogenous Melatonin in Enhancing Salt Tolerance in *Vitis vinifera* L.

**DOI:** 10.3390/ijms25073651

**Published:** 2024-03-25

**Authors:** Qiunan Zhang, Ruiqi Gao, Di Wu, Xiao Wang, Yang Liu, Yanqiang Gao, Le Guan

**Affiliations:** 1 Key Laboratory of Saline-Alkali Vegetation Ecology Restoration, Ministry of Education, Northeast Forestry University, Harbin 150040, China; qiunanzhang1998@nefu.edu.cn (Q.Z.); wd0809@nefu.edu.cn (D.W.); 18438595025@163.com (X.W.); 1127950449@nefu.edu.cn (Y.L.); 2 College of Life Science, Northeast Forestry University, Harbin 150040, China

**Keywords:** melatonin, grape cell cultures, salt stress, transcription regulation

## Abstract

*Vitis vinifera* L. possesses high economic value, but its growth and yield are seriously affected by salt stress. Though melatonin (MT) has been widely reported to enhance tolerance towards abiotic stresses in plants, the regulatory role melatonin plays in resisting salt tolerance in grapevines has scarcely been studied. Here, we observed the phenotypes under the treatment of different melatonin concentrations, and then transcriptome and metabolome analyses were performed. A total of 457 metabolites were detected in CK- and MT-treated cell cultures at 1 WAT (week after treatment) and 4 WATs. Exogenous melatonin treatment significantly increased the endogenous melatonin content while down-regulating the flavonoid content. To be specific, the melatonin content was obviously up-regulated, while the contents of more than a dozen flavonoids were down-regulated. Auxin response genes and melatonin synthesis-related genes were regulated by the exogenous melatonin treatment. WGCNA (weighted gene coexpression network analysis) identified key salt-responsive genes; they were directly or indirectly involved in melatonin synthesis and auxin response. The synergistic effect of salt and melatonin treatment was investigated by transcriptome analysis, providing additional evidence for the stress-alleviating properties of melatonin through auxin-related pathways. The present study explored the impact of exogenous melatonin on grapevines’ ability to adapt to salt stress and provided novel insights into enhancing their tolerance to salt stress.

## 1. Introduction

Grape (*Vitis vinifera* L.) cultivation ranks in the top four in the world by area and is considered a crop of high commercial value by many countries [1,2]. There are more than 70 species of *Vitis*, of which about 38 species are found in China. Grape’s rich nutritional and medicinal ingredients make it a worldwide commodity. The qualities of grape products are characterized by their metabolic compositions, which are composed of primary metabolites (such as fructose and organic acids) and secondary metabolites (such as polyphenols) [1]. The accumulation of metabolites in grapes is limited to a certain extent by salt, water, and temperature [3].

Saline soil is a source of extremely harmful abiotic stress, which prolongs the time of seed germination and plant maturation and reduces the rate of seed setting [4]. Per the data as of 2021, there are millions of hectares of saline soil in China, and the large quantities of soluble salts in saline soil far exceed the range that crops can bear, and the existence of saline soil seems to be more common than ever before [5]. Under salt stress, plant growth, carbon dioxide uptake, and light energy conversion and utilization are all constrained [6], and the diminishment of the electron transport rate and the imbalance of ion homeostasis are exhibited [7]. A high-salt environment can stimulate disorders of a plant’s internal environment. Among these disorders, salt has the most serious negative impact on plants [5]. To minimize damages caused by salt stress, plants launch a series of physiological and biochemical processes, including ion homeostasis and osmotic balance [8]. Elucidating the physiological changes in grapevines under salt stress is crucial for the optimization of berry quality and yields in grape cultivation. 

Melatonin levels vary greatly depending on species and tissue; the amount per gram ranges from a few picograms to several hundred micrograms [9]. Chloroplasts are the main synthesis sites of melatonin, with the mitochondria being secondary sites. Melatonin synthesis is initiated by tryptophan, and six enzymes are involved [10]. Among the six enzymes, L-tryptophan decarboxylase (TDC),Tryptophan 5-monooxygenase (T5H),Arylalkylamine N-acetyltransferase (SNAT),Acetylserotonin O-methyltransferase (ASMT),Catechol-O-methyltransferase (COMT) have been well studied, while Tryptophan hydroxylase (TPH) has been little reported on [11,12]. 

Melatonin has become a well-known substance with many functions, such as regulating the physiological activities and growth of plants [13]. For example, adventitious and lateral root numbers after melatonin treatment in *Arabidopsis* are two times and three times greater in rice [14], respectively. Similar phenomena also occur in tomatoes [15] and cucumbers [16]. Melatonin is not only able to promote plant elongation [12], but it can also improve plant tolerance under abiotic stress [17] and adjust plant vegetative development, such as rooting [15,16], leaf senescence [18], photosynthetic efficient [19], and biomass yield [20]. Furthermore, it has an implied regulatory role throughout the development of flowering plants [21]. In grapevines, melatonin may have a regulatory effect on berry quality by directly regulating glucose metabolism-related pathways and indirectly impacting organic acid accumulation via the regulation of potassium channels in a stressed environment.

The application of plant growth regulators (PGRs) can reduce the yield loss caused by salinity [22]. Nitric Oxide (NO) produced by plants or exogenous NO could reduce salt stress damage [23,24]. Phytohormones play an important role in plant resistance to salt stress. Exogenous gibberellin(GA) treatment could effectively alleviate the effects of salt stress on the growth of sorghum seeds and seedlings [25], and salicylic acid could also improve the damage of rice under salt stress [26]. At low temperatures, melatonin treatment increases plant chlorophyll content and eliminates excessive Reactive oxygen species (ROS) production to promote plant growth [27]. In addition, melatonin promotes the growth of cucumbers [28] and improves the germination characteristics of maize seeds under cold stress [29]. Melatonin treatment increases the germination rate of cotton seeds [30] and regulates carbon and nitrogen assimilation in maize [31] to improve drought tolerance. Melatonin can also alleviate the toxicity of cadmium in tobacco [32]. Exogenous melatonin could improve the salt tolerance of cotton [33,34] and improve the growth of banana seedlings, limonium bicolor, rapeseed seedlings, tea plants, and other plants under salt stress [35,36,37]. Functional studies of melatonin have been reported in other species, while the effects of melatonin on the growth of grapes under salt stress need to be further explored. 

Auxin is present almost throughout the entire life course of plants [38], especially under abiotic stresses [14,39]. Auxin induces the rapid expression of auxin/indo-3-acetic acid (*Aux*/*IAA*), Gretchen Hagen 3 (*GH3*), and *small auxin-UPR* (*SAUR*). Aux/IAA directly regulates auxin signaling as a transcriptional repressor, and auxin signaling helps plants resist stressful environments. The expression levels of *OsIAA2* and *OsIAA20* in rice are increased under the induction of salt stress [40,41]; *VvIAA* overexpression enhances the salt tolerance of tobacco [42]. Under drought stress, *IAA* in *Arabidopsis* is up-regulated [43]. *OsIAA18* overexpression increases the salt tolerance threshold of the plant and improves the plant ability to repair osmotic disorders [44,45]. Genes involved with the increment of auxin levels facilitate resistance to adverse environments in tomatoes, poplars, and so on. For example, *YUCCA6* has the power to up-regulate IAA content [46,47]; this plays a role in abiotic stress, and *YUCCA6*-overexpressing plants show higher stress tolerance [48].

Compared with traditional whole plant cultures, cell cultures are easy to manipulate, save costs and labor, and can be continuously maintained independent of the growing seasons. Cell cultures are also the main raw material of suspension cells. Suspension cells are widely used in large-scale industrial production. In our paper, 1 and 100 μM of melatonin were added to the solid medium to explore the effect of melatonin on metabolite changes. To elucidate the roles melatonin plays in grapes coping with salt stress, the cell cultures were simultaneously treated with melatonin and NaCl. The transcriptomic analysis was performed to reveal the molecular basis. This deepened our understanding of melatonin and could provide a potential strategy to increase berry quality under salt stress.

## 2. Results

### 2.1. The Analysis of Metabolites and Transcript Levels under Melatonin Treatment

**Figure 1:**
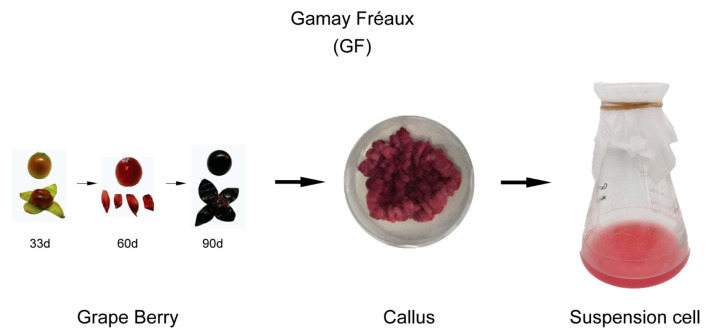
The phenotype of ‘Gamay Fréaux’ berry at 33, 60, and 90 days after anthesis. The cell cultures were derived from the berry pulp 90 days after anthesis, and then the cell cultures could be further cultivated into a cell suspension.

**Figure 2:**
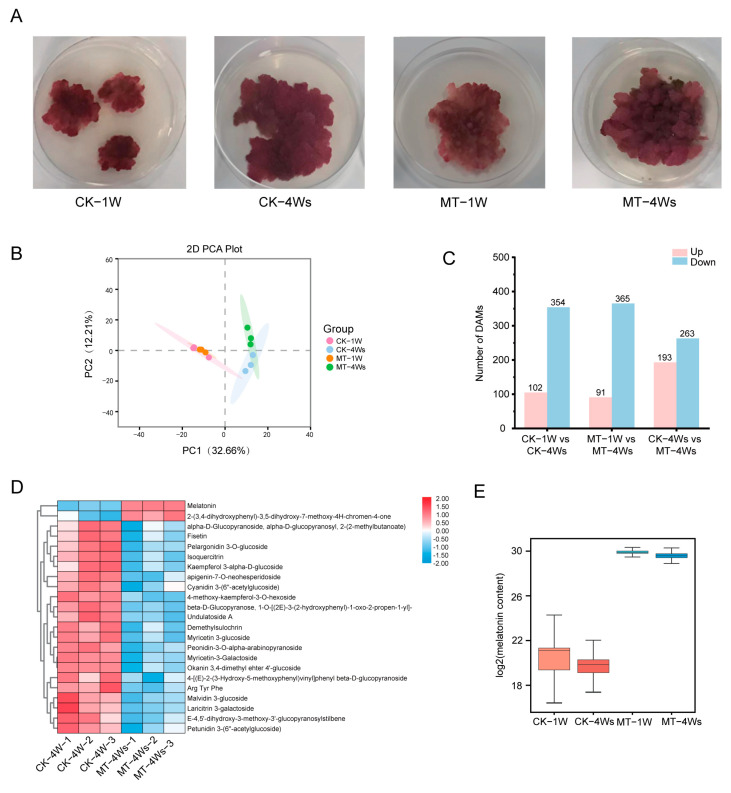
(**A**) Effect of melatonin treatment on grape cell cultures with different growth weeks: 1W, 1 week after treatment; 4Ws, 4 weeks after treatment; MT, melatonin. (**B**) PCA, principal component of metabolites. PC1 and PC2 are the first and second principal components. (**C**) Several differentially accumulated metabolites (DAMs). (**D**) Heatmap of metabolites using log2-fold change. (**E**) Change of melatonin content.

Cell cultures were derived from Gamay Fréaux berries 90 days after anthesis. The grape cells in good growth conditions were used as the experimental materials (Figure 1). The appearance of the cell cultures treated with the concentrations of 1 μM (MT1) and 100 μM (MT100) of melatonin were less dark than the CK (without melatonin applications) (Figure 2A). Cell cultures in good growth conditions at 1 week after treatment (1W) and 4Ws were used for sequencing. 

**Figure 3:**
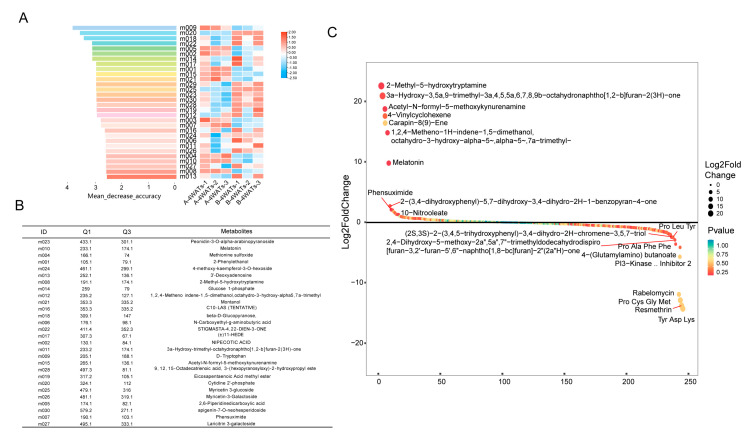
(**A**) Random forest analysis and (**B**) annotated information for the top 30 metabolites. (Q1, molecular weight; Q3, maximum parent nucleus mass). (**C**) Dynamic distribution of metabolite content differences.

There were 457 metabolites detected in both the treated and untreated cell cultures at 1 and 4 weeks after treatment. The Principal Component Analysis (PCA) showed that samples from each treatment were tightly clustered. Principal Component 1 (PC1) was well separated during the developing stages while Principal Component 2 (PC2) to a certain extent separated from the melatonin treatment at 4Ws (Figure 2B). Pairwise comparisons were further performed between samples at different developmental stages and melatonin concentrations (Figure 2C). There were more down-regulated metabolites (354, 365, and 263) than up-regulated ones (102, 91, and 193) in CK-1W vs. CK-4Ws, MT-1W vs. MT-4Ws, and CK-4W vs. MT-4Ws. Metabolite changes in the pairwise comparison between CK-4Ws and MT-4Ws were further studied in detail. The increment in the endogenous melatonin and the decreases in 21 compounds mainly including flavonoids were demonstrated (Figure 2D). After melatonin treatment, there was a clear increase in endogenous melatonin content, from 20 to 30, which increased approximately 1.5-fold (Figure 2E). The differential metabolites of CK-4W vs. MT-4W were arranged according to their contribution (Figure 3A), and their expression levels were significantly different among the four treatment groups. The names and masses of metabolites are listed (Figure 3B). A metabolite ranking dot plot screened the top and bottom 10 metabolites, which supported the reliability of the metabolites selected by random forest analysis (Figure 3C). The Venn diagram indicated that there were 21 common metabolites between the melatonin-treated and untreated cultures (Figure S1).

**Figure 4:**
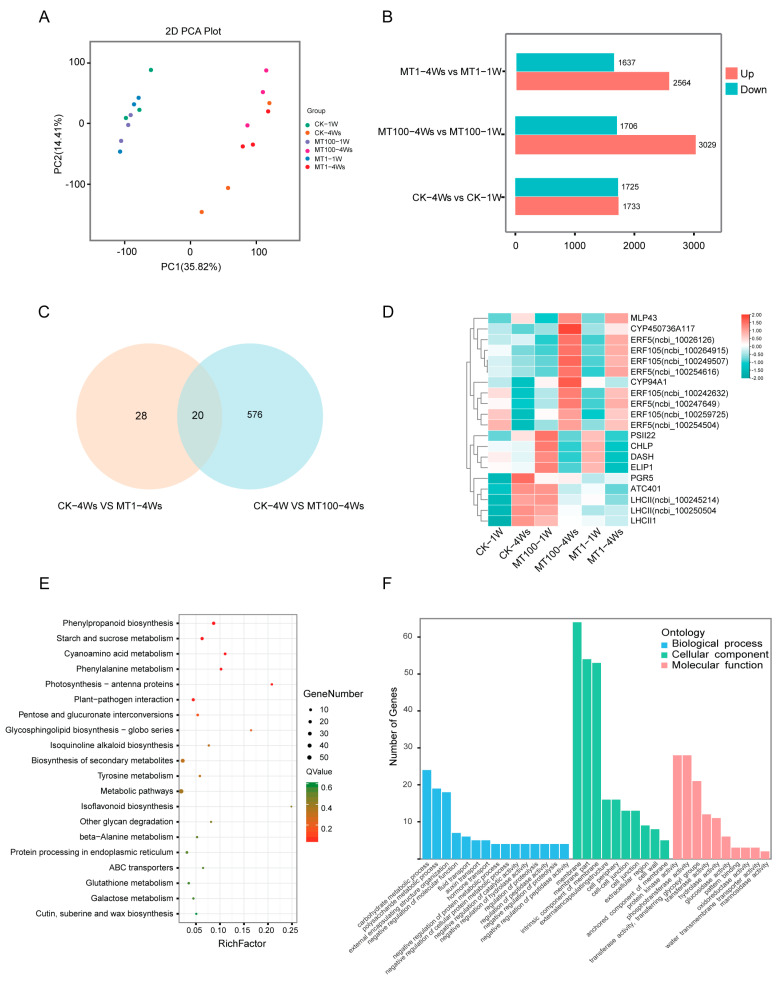
(**A**) PCA (principal component analysis) results based on all transcripts identified by RNA-seq. (**B**) Several differentially expressed genes. (**C**) Venn figure shows the differential genes of CK-4Ws vs. MT1-4Ws and CK-4Ws vs. MT100-4Ws, and the (**D**) expression of 20 genes is cross-focused. (**E**) KEGG and (**F**) GO of CK-4Ws and MT100-4Ws of DEGs.

The metabolism data suggested significant changes in the melatonin and flavonoid levels under the melatonin treatment. To explore the potential regulatory mechanism at the transcriptional level, we conducted a transcriptome analysis. The transcriptome analysis detected a total of 27,176 different expressed genes (DEGs). The samples correlated well among replicates and were well-distinguished among the samples (Figure 4A). the number of genes with raised expressions was significantly increased with the melatonin concentrations, from 1733 (0 μM) to 3209 (100 μM) (Figure 4B). The quantitative differences in the DEGs between 1 and 100 μM of melatonin treatment (Figure 4C) indicated that only 28 genes were differentially expressed under 1 μM of melatonin treatment, while 576 genes under 100 μM of treatment. There were 20 genes common to both MT1 and MT100, and the heatmap applied the expression of them (Figure 4D). A total of eight ERF (ethylene responsive factor) genes were up-regulated by melatonin treatment. CK-4Ws vs. MT100-4Ws, which showed the most obvious response, were subjected to KEGG (Figure 4E) and GO enrichment analyses (Figure 4F). The pathways related to flavonoid metabolism (“phenylpropanoid biosynthesis”, “phenylalanine metabolism”, and “isoflavonoid biosynthesis”), sugar metabolism (“starch and sugar metabolism”, “pentose and glucuronate interconversions”, and “galactose metabolism”), “photosynthesis-antenna proteins”, and “ABC transporter proteins” were significantly enriched (Figure 4E). The GO enrichment analysis functionally characterized the DEGs into 35 functional groups (Figure 4F), of which biological processes (BPs) contained 15 groups and cellular components (CCs) and molecular functions (MFs) contained 10 groups each. In the BPs, the “carbohydrate metabolic process”, “polysaccharide metabolic process”, “negative regulation of molecular function”, “auxin transport”, and “hormone transport” had a high number of transcripts.

### 2.2. The Correlation Network under Melatonin Treatment

**Figure 5:**
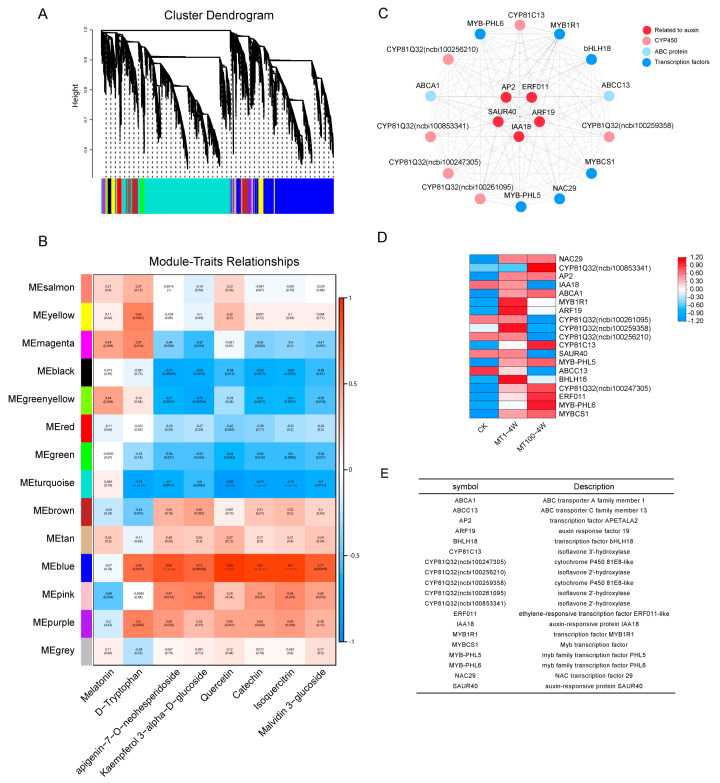
(**A**) Hierarchical clustering trees and modules for all differentially expressed genes. Different colors represent different modules. (**B**) The co-expression networks of module-trait-related genes during melatonin treatment and growth were analyzed by WGCNA. Module–trait correlations are shown, and the corresponding *p*-values are shown in parentheses. (**C**) Correlation network diagram of HUB gene in MEblue module relative to auxin gene expression heatmap (**D**) and (**E**) functional annotation.

To further understand the regulation rules of metabolite changes during grape development and the response to the melatonin treatment, WGCNA was performed to investigate the common networks (Figure 5A). Based on the random forest analysis, seven metabolites, including different types of flavonoid substances (flavones, flavanones, flavanols, and anthocyanins) as well as melatonin and the melatonin precursor tryptophan, were selected as traits, and the key modules and genes were identified by WGCNA (Figure 5B). The MEblue module exhibited notable positive correlations (R^2^ ≥ 0.68) with all the seven metabolites except melatonin. The MEmagenta module manifested a significant positive correlation with melatonin and tryptophan, with the correlation coefficients being 0.44 and 0.57. A detailed description of modules and trait correlations (Figure 5B).

To further explore the candidate genes with significant contributions to gene networks, all genes in the MEblue and MEmagenta modules were extracted to visualize networks (Figure 5C). The screening of genes in the MEblue module identified 20 genes, including six cytochrome oxidase genes (one *CYP81C13* and five *CYP81Q32*), four MYB transcription factors (*MYB-PHL5*, *MYB-PHL6*, *MYB1R1*, and *MYBCS1*), four genes responses to auxin (*AP2*, *ARF19*, *IAA18*, and *SAUR40*), two bHLH transcription factors (*bHLH18* and *bHLH*), two ABC transporter genes (*ABCA1* and *ABCC10*), one ethylene-responsive transcription factor (*ERF011*), and one NAC transcription factor (*NAC29*). The heatmap shows the expression of all the genes in the network (Figure 5D). The expression levels of the three CYPs changed with an increase in melatonin concentration, and the other three showed the opposite trend. Auxin response genes were down-regulated expressions in MT1, but up-regulated in MT100. As the concentration of melatonin increased, *ABCA1* and *ERF011* were up-regulated, and *ABCC10* was down-regulated (Figure 5E).

### 2.3. Effects of Melatonin Treatment upon Salt Stress of ‘Gamay Fréaux’ Grape Cell Cultures

Based on the above results, melatonin treatment modified the genes associated with resistance to salt stress. So, the cell cultures were simultaneously treated with melatonin and NaCl to explore the roles melatonin play in salt stress.

#### 2.3.1. Phenotypic Observations 

**Figure 6:**
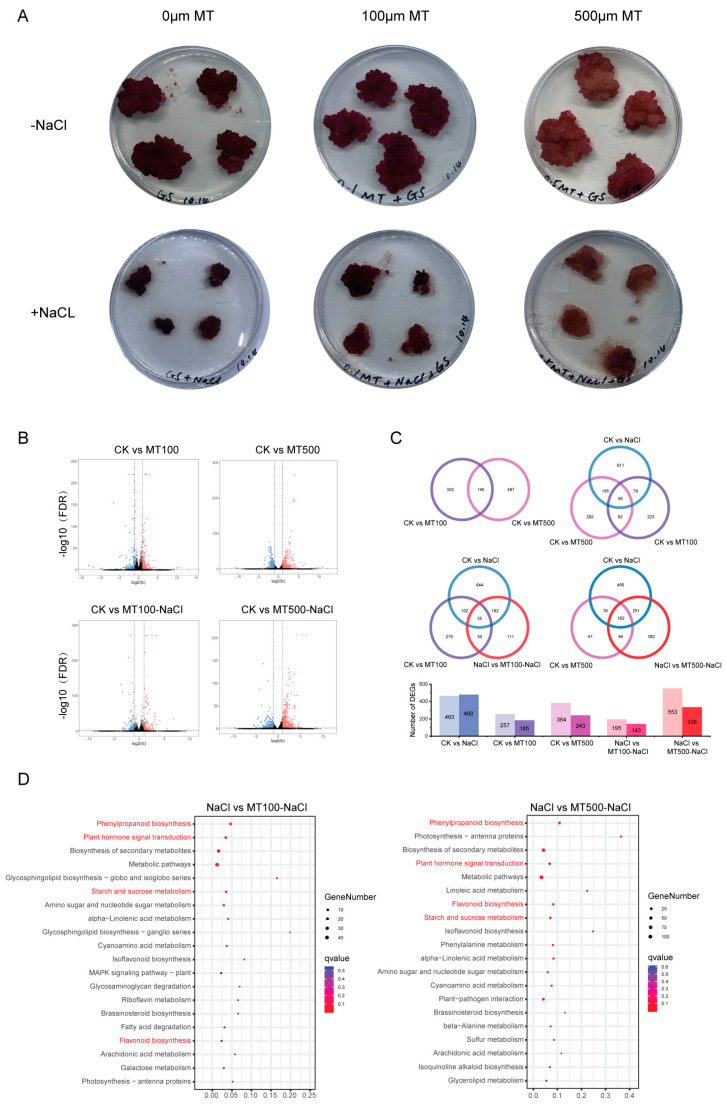
(**A**) Phenotypic changes of grape cell cultures treated with melatonin and NaCl. (**B**) Volcanic map of differentially expressed genes in grape cell cultures under melatonin and NaCl. (**C**) The Venn diagram shows the number of different genes in different groups. (**D**) KEGG analysis of differential genes between NaCl vs. MT100-NaCl and NaCl vs. MT500-NaCl groups.

Compared with CK, the cell cultures were looser, and the color was more purplish for MT100 and MT500. Under NaCl treatment, the growth of cell cultures was significantly inhibited, and the cells were dried and turned brownish. MT100-NaCl alleviated the salt stress to some extent since the cells became less brown and dried when compared with the NaCl treatment. The cell cultures for MT500-NaCl appeared light purplish–red with no obvious dehydration (Figure 6A).

#### 2.3.2. The Transcriptomic Analysis

All 1069 DEGs were detected in the melatonin treatment groups: 442 (257 up and 185 down) in CK vs. MT100 and 627 (384 up and 243 down) in CK vs. MT500, while for 1227 DEGs detected in the melatonin + NaCl group: 338 (195 up and 143 down) in NaCl vs. MT100-NaCl and 889 in NaCl vs. MT500-NaCl (553 up and 336 down) (Figure 6B). There were 140 common genes between MT100 and MT500, and MT500 showed more differential genes when compared with CK (the left upper panel). In melatonin and NaCl treatment, there were only 58 common genes, and more DEGs responded to the salt stress than they did to melatonin treatment (upper right panel). When taking both salt stress and melatonin treatment into consideration, there were more common genes at higher melatonin concentrations: 35 at MT100 and 182 at MT500. In addition, more DEGs were detected when treated with higher melatonin concentrations (MT500) upon salt stress (lower panel, Figure 6C). 

To further explore the DEGs in NaCl vs. MT100-NaCl and NaCl vs. MT500-NaCl, pathways were enriched by KEGG (Figure 6D). The top 20 enriched pathways included secondary metabolism (“phenylpropanoid biosynthesis”, “biosynthesis of secondary metabolites”, “metabolic pathways”, “phenylpropanoid biosynthesis”, “isoflavonoid biosynthesis”, “riboflavin metabolism”, and “flavonoid biosynthesis”), primary metabolism, especially sugar metabolism (“Starch and sucrose metabolism” and “galactose metabolism), “plant hormone signal transduction”, etc. The enrichment degree of “flavonoid biosynthesis”, “starch and sucrose metabolism”, and “phenylpropanoid biosynthesis” pathways in NaCl vs. MT500-NaCl was slightly higher than that in NaCl vs. MT100-NaCl.

**Figure 7:**
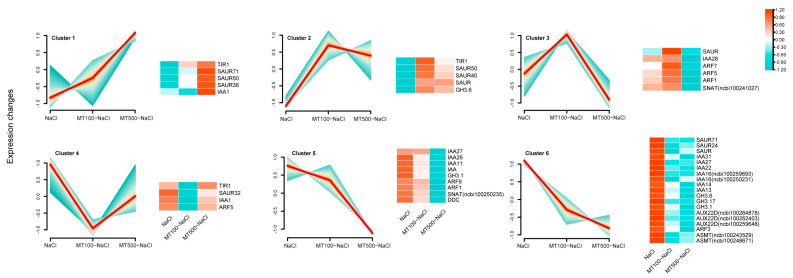
Cluster of all genes and heatmap of genes related to melatonin and IAA synthesis among them.

All DEGs were divided into six parts according to their expression trends in NaCl, MT100-NaCl, and MT500-NaCl (Figure 7). The six clusters could be further grouped into four categories. Cluster 1 showed continuous increasing expression (*SAUR*), while clusters 5 and 6 exhibited a steady decline (*IAAs*, *AUXs*, *GH3*, *ARFs*, *SNAT*, and *ASMT*). Clusters 2 and 3 peaked under MT100-NaCl (*SAUR, ARF*, and *SNAT*), whereas cluster 4 displayed the lowest point under MT100-NaCl.

#### 2.3.3. The Correlation Network Analysis

**Figure 8:**
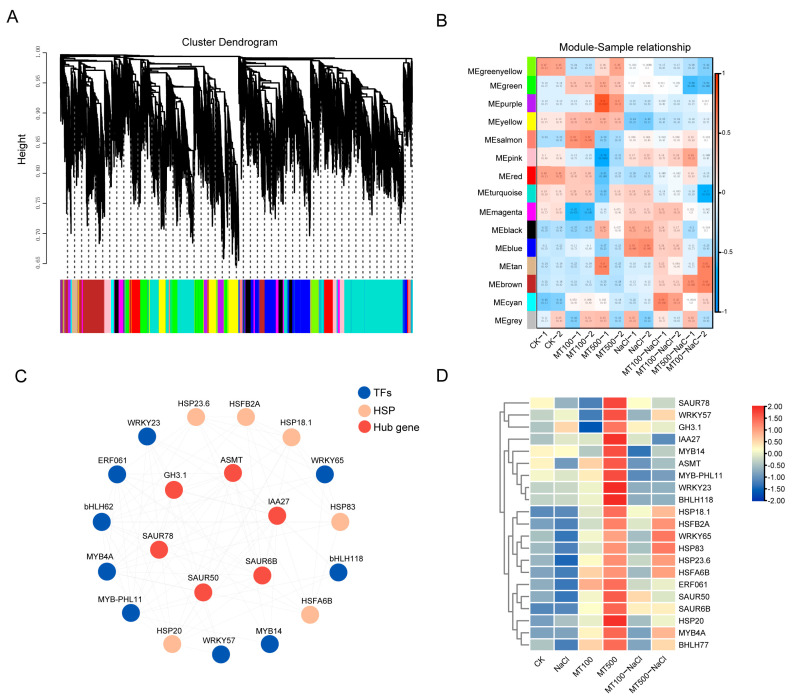
(**A**) WGCNA of differentially expressed genes screened from grape cell cultures treated with NaCl and melatonin. (**B**) Module–trait relationships. Each cell contains the corresponding correlation and *p*-value. Red and blue represent positive and negative correlations, respectively, and the darker the color, the stronger the correlation. (**C**) Gene connectivity network. (**D**) Gene expression heatmap in the MEpurple module.

The DEGs’ common network of melatonin-treated groups was analyzed through WGCNA (CK, MT100, and MT500) and salt-melatonin-treated groups (NaCl, MT100-NaCl, and MT500-NaCl) (Figure 8A). A total of 15 gene expression modules were identified, and the following five modules were selected according to the module-gene correlation heatmap. MEsalmon was positively correlated with CK vs. MT100; MEmagenta and MEcyan were positively correlated with NaCl vs. MT100-NaCl; MEbrown was positively correlated with MT500 vs. MT500-NaCl, while MEpurple was negatively correlated with it (Figure 8B). MEpurple was further analyzed as the key module, and 25 genes were listed as genes associating with salt stress under melatonin treatment, among which four genes were related to auxin signal transduction (*IAA27*, *SAUR78*, *SAUR50*, and *GH3*), one was a melatonin-synthesis-related gene (*ASMT*), and nine were transcriptional factors (Figure 8C). Under NaCl, their expression decreased, while being up-regulated by melatonin treatment, with extraordinarily high expression under MT500, followed by MT500-NaCl and MT100-NaCl (Figure 8D). 

##### 2.4. qPCR Validation

**Figure 9:**
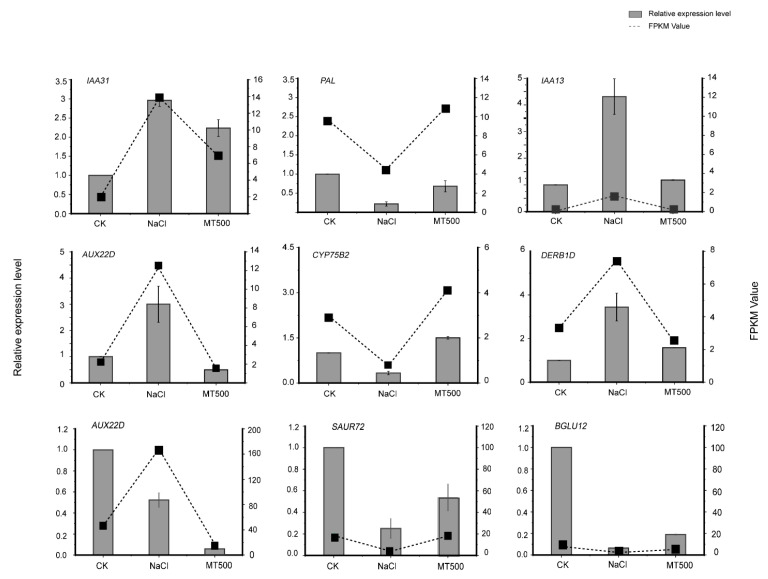
Expressions of *IAA31, PAL, IAA13, AUX22D, CYP75B2, DREB1D, AUX22D, SAUR72,* and *BGLU12* in grapes were examined by qRT-PCR analysis and FPKM values.

The 14 DEGs among CK, MT100, and MT500 within high KEGG enrichment pathways were randomly selected to validate the transcript. The results showed that the relative expression levels of nine genes in qPCR were consistent with the detection of the transcriptome (Figure 9).

## 3. Discussion

The grape has extremely high economic value. It’s subsidiary products have good commercial prospects and markets, but soil salinization seriously limits the normal life course of grapes [1,2]. Melatonin is an important biological molecule that interacts with a variety of substances during plant development, especially to help regulate adaptive plant responses to abiotic stresses. Existing studies suggest that melatonin treatment can enhance plant salt tolerance in tomatoes [3], apples [4], barley [5], and rice [6]. Melatonin treatment mediates tolerance to salt in cotton by adjusting Ca^2+^ and reactive oxygen scavenging mechanisms [7]. Exogenous melatonin treatment could alleviate the salt stress of rice seedlings by the improvement of leaf photosynthesis [8]. There is new evidence that melatonin spraying promotes grape berry growth and enlargement, increases the content of anthocyanins in grapes, and promotes fruit ripening [49]. To elucidate how melatonin functions under salt stress, metabolomic and transcriptomic analyses were conducted when the cell cultures were simultaneously treated with NaCl and melatonin (100 and 500 μM) in the present study. Exogenous melatonin could promote the synthesis of endogenous melatonin and auxin, thereby increasing the salt tolerance of grapes [16]. This is consistent with the results of the present study. 

Melatonin promotes endogenous melatonin synthesis to maintain fruit quality by up-regulating gene expressions in the melatonin biosynthesis pathway [4]. In our study, the melatonin content of grapes was raised more than hundreds of times under exogenous melatonin treatment, with more than 500 DEGs responding to MT100 of melatonin treatment (Figure 2C–E). Transcriptome analysis showed that 100 μM of melatonin treatment had more differentially expressed genes than did 1 μM of melatonin, which suggested that the 100 μM treatment might have had a more obvious effect. The DEGs were enriched in benzene–propane synthesis and sugar and starch metabolism. GO analysis displayed that 5% of the genes were related to auxin transport and 5% of the genes were related to hormone transport. WGCNA showed some key genes, *IAA18*, *SAUR40*, *ARF19*, *AP2*, *ERF011*, and ABC transporter families, were all related to auxin signal transduction (Figure 5C). Previous studies have demonstrated that melatonin treatment increases the level of auxin to enhance auxin signaling [50]. Melatonin has synergistic or antagonistic effects with other hormones. As a plant growth regulator, 100 μM of melatonin can promote the growth of roots and young leaves of plants [26], and IAA also has similar effects. Combined with our results, this suggested that the accumulation of melatonin has a positive influence on the normal growth of plants and can cooperate with auxin to promote plant growth. We also suggest that melatonin treatment may affect plant metabolism, internal transport, elongating, and thickening, dealing with tolerance and other processes. But this is not well studied. Under stress conditions, the expression of melatonin-synthesis-related genes increases, and more melatonin is produced to adapt to stress changes, especially in a high salt environment [9,10,11,12]. Not only melatonin, but also melatonin precursors, intermediates, and metabolites are present in plant responses to stress [11,16]. Tryptophan (TRP), a precursor to auxin, indole, alkaloids, and phytoalexins, is an important plant stress response substance. Tryptamine and serotonin have also been found in plants to resist abiotic stresses [13,14], and based on their close correlation with melatonin, many substances in the melatonin synthesis pathway have been proposed to be related to stress resistance.

**Figure 10:**
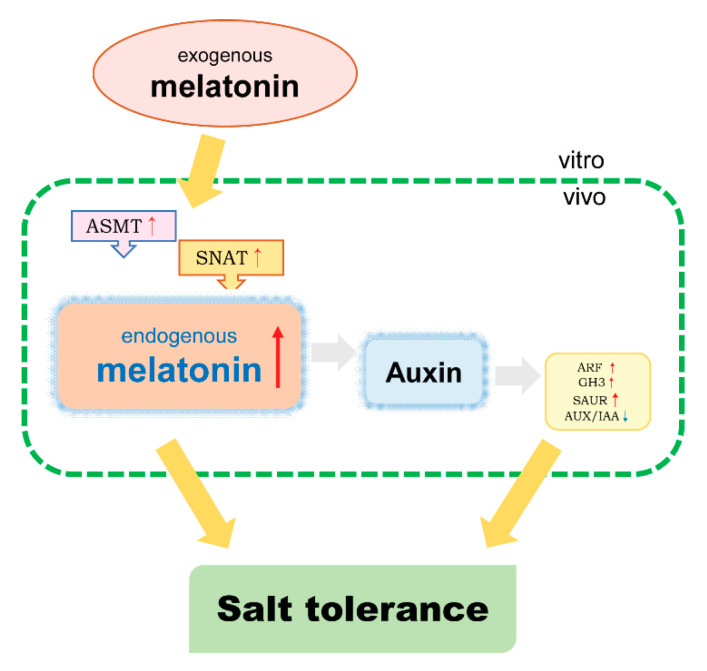
Exogenous melatonin-treatment-induced changes in the expression of melatonin synthesis genes *SNAT* and *ASMT*, which directly led to significant changes in endogenous melatonin content. Melatonin increases auxin activity, and auxin promotes the expression of response genes *ARF*, *GH3*, up-regulated *SAUR*, and down-regulated *AUX/IAA*. Melatonin synthesis and auxin signal transduction can mediate salt tolerance in grape cell cultures. In the figure, gray arrows indicate results reported in the previous literature, yellow arrows indicate results obtained in this paper, red arrows indicate up-regulated gene expression, and blue arrows indicate down-regulated gene expression.

*SNAT* and *ASMT* were up-regulated under salt treatment, while decreased under melatonin and salt, and 500 μM of melatonin decreased their expression the most (Figure 7). This meant that, additionally, melatonin may share tasks with endogenous melatonin. Studies have shown that *VvIAA18* overexpression, and *SAUR41* subfamily expressions (*SAUR40*, *SAUR41*, *SAUR71*, and *SAUR72*) regulate salt tolerance [13,14,15]. During our research, up-regulated expressions of *IAA27*, *SAUR78*, *SAUR50*, *SAUR6B*, and *GH3* were detected after melatonin treatment. We did phenotypically observe that melatonin did alleviate the effect of salt, and the effect of 500 μM of melatonin treatment was significantly better than that of 100 μM. Combined with the above research and existing reports, a hypothesized model of the salt tolerance mechanism of grape cell cultures mediated by melatonin under salt stress was developed (Figure 10). On the one hand, melatonin enhanced plant salt tolerance by increasing endogenous melatonin content and regulating the melatonin-synthesis-related genes. On the other hand, melatonin increased auxin biosynthesis and signal transduction, which may have reduced the damage of the high salt environment to the body and maintained normal physiological activities through changes in the auxin signaling pathway. 

Previous studies have reported melatonin mainly as a growth promoter and antioxidant, with biological activities such as delaying aging, enhancing photosynthesis, regulating photoperiods, and affecting seed germination and root morphogenesis [51]. It can also effectively improve plant stress resistance and is conducive to the survival and reproduction of plants [12]. Our country is rich in grape germplasm resources. To make full use of our excellent genetic resources, and in-depth study of grape responses to adverse environments is of great importance. Understanding the regulation mechanism of important salt tolerance pathways and genes is of great significance for a comprehensive understanding of the nature of grape adaptation to salt stress. The addition of melatonin is a potential strategy to maintain the normal growth and quality of grapevine, and the present study provides information regarding its potential regulatory network at the transcriptional level. This is important for promoting the development of grape and grape-affiliated industries and can be widely used in production practices in the future.

## 4. Materials and Methods

### 4.1. Plant Materials

“Gamay Fréaux” cell cultures were generously provided by Dr. Serge Delrot’s group (EGFV, ISVV, INRA). The cell cultures were cultivated in Murashige & Skoog medium (Duchefa, Biochemie B.V, Haarlem, The Netherlands), supplemented with 20 g/L sucrose(solarbio, Beijing, China), 0.1 mg/L NAA (promise biochemical, Beijing, China), 0.2 mg/L KT (BBI, Shanghai, China), and 0.8% agar (solarbio, Beijing, China) at 22 ± 2 °C with continuous illumination. An amount of 0.1 M KOH was added to the medium until the pH was 5.8 before autoclaving. The cell cultures were transferred to a new solid medium in 28-day cycles. The purplish–red tissue with loose cells was selected as the experimental material.

### 4.2. Sample Treatment and Collection

To explore what changes occurred in grape cell culture growth and metabolism following melatonin treatment, the cell cultures were treated with concentrations of 1 μM (MT1) and 100 μM (MT100) of melatonin, and then were separately collected at one week after treatment (1 WAT, MT1-1W, MT100-1W) and four weeks after Gamay Fréaux treatment (MT1-4Ws, MT100-4Ws). The cell cultures without melatonin applications were referred to as CK (CK-1W and CK-4Ws). Furthermore, to investigate the roles melatonin plays in salt stress, the cell cultures were simultaneously treated with 0.8% sodium chloride (NaCl) and two concentrations of melatonin: 100 and 500 μM. In detail, CK was a blank control without melatonin and NaCl applications, “NaCl” was treated with NaCl, MT100 and MT500 were separately treated with melatonin of 100 and 500 μM, and MT100-N and MT500-N were treated with melatonin of the two concentrations mentioned above and 0.8% NaCl. Different concentrations of melatonin and 0.8% NaCl were added in the media at the start of the culture. The cell culture sample was collected at 2 WsAT without any touch of the media. Grape cell cultures were briefly treated with liquid nitrogen and stored at −80 °C for subsequent analysis. Each independent cell mass was maintained in triplicate.

The samples were freeze-dried under a vacuum. The dried samples were ground to a powder using a mortar. The 100 mg powder sample was dissolved in 70% methanol containing 0.1 mg/L lidocaine as an internal standard, centrifuged at 10,000× *g* for 10 min at 4 °C overnight, and the precipitate was discarded and filtered through a 0.22 μM microporous filter membrane.

Chromatographic column: the waters ACQUITY UPLC HSS T3 C18 (1.8 μM, 2.1 mm × 100 mm) were maintained at a column temperature of 40 °C. Water containing 0.04% formic acid was mobile phase A, and acetonitrile containing 0.04% formic acid was mobile phase B. The elution gradient was as follows: 0 min, 5% B; 11 min, 95% grade B; 12 min, 95% grade B; 12.1 min, 5% B; API 6500 QTRAP LC/MS/MS operated in both positive and negative ion modes to analyze the eluted metabolites. Ionization source condition: a temperature of 550°C; a 5500 V spraying voltage; 25 psi curtain gas. Metabolites were separately detected in positive and negative ion modes.

### 4.3. RNA Extraction, Library Construction, and Sequencing

A Trizol kit was used to extract the total RNA from the samples. RNA quality and purity were assessed using an Agilent 2100 Bioassay system, and RNA bands were detected by agarose gel electrophoresis. After the RNA was obtained, eukaryotic mRNA was enriched using Oligo (dT) magnetic beads, and rRNA was removed using a Ribo-Zero TM magnetic kit. In the fragmentation buffer, the mRNA fragments were fragmented into several small fragments. The final cDNA was obtained by reverse transcription of these small fragments with random primers. After end repair and polyA tail addition, the purified cDNA fragments were screened by agarose gel electrophoresis, amplified by PCR, and sequenced on Illumina HiSeq2500.

### 4.4. Transcriptome Analysis

Transcriptome and metabolome data were obtained from GENE DENOVO Biotechnology Co., Ltd. (Guangzhou, China). The offline data were screened again by fast (version 0.18.0). Clean dates were aligned to the reference genome by HISAT2.2. The mapped sequence of each sample was assembled according to the reference method, which was done by StringTie (version 1.3.1), and the FPKM value of each transcribed region was calculated. DESeq2 software (version 3.4.1) was used to analyze the differential expression of RNA between the two groups. The false discovery rate (FDR) of discrepantly expressed gene transcripts was <0.05, and the absolute fold change was ≥2. PCA was performed using R (http://www.rproject.org/, accessed on 1 November 2023).

### 4.5. Quantitative PCR (qPCR) Assay

To verify the reliability of the transcript, qPCR was performed, which was performed with the same samples as those used for RNA sequencing. A TOYOBO ReverTra Ace^®^qPCR RT Kit was used for qPCR. The expressed level was analyzed in the Roche Light Cycle 480 system. The primers of the genes were designed by SnapGene and verified by NCBI. ACTIN was selected as the reference gene, and the relative gene expression was analyzed by the 2^−∆∆Ct^ method. 

### 4.6. Statistical Analyses

Analyst (version 1.6.1) was used for data filtering, peak detection, alignment, and calculation. The filtered data were uploaded to the R package (http://www.r-project.org/), and the metabolites from each sample were stratified for clustering analysis. Substances with significantly different expression levels in each group were filtered. The screening threshold of the DAMs was a VIP ≥ 1, calculated with a T-test (*p* < 0.05). The metabolomic analysis was performed for three replicates. Statistical analyses of significant differences were evaluated by one-way analyses of variance (ANOVA), followed by Tukey’s HSD test. All statistical tests considered *p* < 0.05 to be statistically significant. PCA, boxplot, random forest, WGCNA, and volcano maps were produced using an online platform https://cloud.metware.cn/ (accessed on 1 November 2023). The histogram was drawn with Origin2023. The Venn diagrams and heatmaps were produced using TBtools. Gene cluster trends were completed with the online site Hiplot https://hiplot.cn/ (accessed on 20 November 2023). The metabolite rank plot was plotted using the online site Tomicsvis Cloud https://github.com/cran/TOmicsVis (accessed on 15 November 2023). The network regulation diagram was drawn with Cytoscape (version 3.10.1).

## 5. Conclusions

In summary, exogenous melatonin significantly up-regulated the expression of genes related to melatonin synthesis, leading to a higher accumulation of endogenous melatonin. Phenotypically, the adverse effects of salt stress on cell cultures could be effectively attenuated by exogenous melatonin treatment. Further, transcriptome analyses revealed that exogenous melatonin enhanced grape salt tolerance by regulating the expression of genes that participate in melatonin synthesis and the signal transduction of auxin. Overall, this study helps to further expand our understanding of grape salt tolerance and has important implications for changing the current limitations of the grape industry in high-salt land.

## Data Availability

The data that support the findings of this study are available from the corresponding author upon reasonable request.

## Supplementary Materials

The following supporting information can be downloaded at: https://www.mdpi.com/article/10.3390/ijms25073651/s1.
