# Supplementary material for: Metabolome and Transcriptome Analysis Revealed the Pivotal Role of Exogenous Melatonin in Enhancing Salt Tolerance in Vitis vinifera L
Source: Int J Mol Sci. 2024 Mar 25;25(7):3651. doi: 10.3390/ijms25073651 (PMC11011403; doi:10.3390/ijms25073651)
Supplement: Supplementary file 1 [file ijms-25-03651-s001.zip › Supplement Table S1.pdf]

Supplement table. primers list for qPCR

Gene	F/R	Sequence
Actin	F	CTTGCATCCCTCAGCACCTT
	R	TCCTGTGGACAATGGATGGA
IAA31	F	CCACATTCATCGTCATCATC
	R	CGAATAAGTTAGTGGCTTGG
PAL	F	TTAATCTCCTCCCGGAAAAC
	R	GGTCATCGATGTAGGCAAAT
IAA13	F	ATGGATAGTGGTCTGAGTTC
	R	CGGAAGAATTAGAGAAGCGA
AUX22D(Center left)	F	GGAGAACAAGGTCATATACG
	R	GCTTTTCCGGTATGATCGAA
DREB1D	F	TCCTGGCACTGGCAGCACGTG
	R	GAGCGGAAAGTGAAGCACTGA
CYP75B2	F	CCGCTCAGTTCTTGAAAACT
	R	GGATATGGCGAAAATCATCC
BGLU12	F	GAAAGGAATGAGCTTGGATGC
	R	TCACCCTATCCCCAAATTCT
AUX22D(Bottom left)	F	AAGGGAAGTTCTAATGGTGA
	R	ATATTCTGTAGAGCCTGAAG
SAUR72	F	ATGAAGAAGTTGATCCGC
	R	AAGACCGGGTGGTTAAGAAA
