# Supplementary figures and images for: Metabolome and Transcriptome Analysis Revealed the Pivotal Role of Exogenous Melatonin in Enhancing Salt Tolerance in Vitis vinifera L
Source: Int J Mol Sci. 2024 Mar 25;25(7):3651. doi: 10.3390/ijms25073651 (PMC11011403; doi:10.3390/ijms25073651)

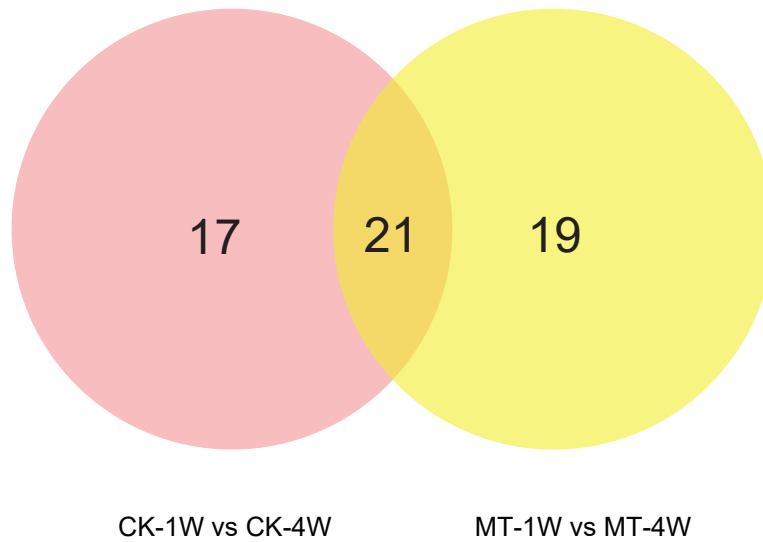

Figure S1. Venn diagrams show the number of DEGs in CK-1W vs CK-4W and MT-1W vs MT-4W.

Supplement: Supplementary file 1 [file ijms-25-03651-s001.zip › Supplement Figure S1.pdf]

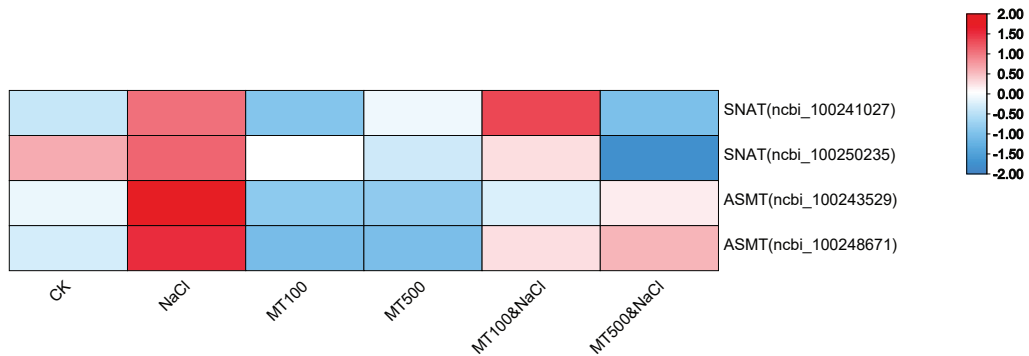

Figure S2. Heatmap of melatonin synthesis genes in CK, NaCl, MT100, MT500, MT100-NaCl, and MT500-NaCl.

Supplement: Supplementary file 1 [file ijms-25-03651-s001.zip › Supplement Figure S2.pdf]
